# 

**Published:** 1878-11-20

**Authors:** 


					﻿VOL. VIII.	NOV. SO, 1878.	No. 11.
ft
W
04
8
H
W
04
-<
V>
PH
Q
W
S
04
P
O
O
H
i-3
5K
04
t>
O
*—5
cn
ta
o
vy
5
t—i
-<
J
O
M
tt
H
H
a
04
0-
w
0D
■<
K
tJ
6
THE SOUTHERN
MEDICAL RECORD.
^LONTHLY jloU£\NAL OF PRACTICAL ^.EDICINE.
Terms : 82 OO per annum In advance. Club Rates, 5 copies
S8.OO. Single copies 20 cents.
“QUICQUTD PRJECIJPIES ESTO BREVIS.”
. 1 •
S2
W
Q
w
&
a
2
i—i
Q
►
4
o
OD
U
►a
to
p>
3
t—(
o
f
Ho
H
W
5
00
OD
O
t“*
h-<
Q
i—(
W
o
15
____________ESTABLISHED ITJ 1810.
ATLANTA, GA.:
Sunnt South Steam Poblmhimg House.
Address R. C. WORD, M. D., Managing Editor, Atlanta, Ga.
				

